# Prevalence and risk factors of type-2 diabetes mellitus in Ethiopia: systematic review and meta-analysis

**DOI:** 10.1038/s41598-021-01256-9

**Published:** 2021-11-05

**Authors:** Melkamu A. Zeru, Endalamaw Tesfa, Aweke A. Mitiku, Awoke Seyoum, Tesfaye Abera Bokoro

**Affiliations:** 1 Department of Statistics, College of Science, Bahir Dar University, Bahir Dar, Ethiopia; 2 Department of Biochemistry, College of Medicine and Health Science, Bahir Dar University, Bahir Dar, Ethiopia; 3 Department of Statistics, College Computing and Informatics, Haramaya University, Dire Dawa, Ethiopia; 4 School of Mathematics, Statistics and Computer Science, College of Agriculture Engineering and Science, University of KwaZulu-Natal, Durban, South Africa

**Keywords:** Health care, Risk factors

## Abstract

Diabetes mellitus (DM) is a public health problem in developing as well as developed nations. DM leads to many complications that are associated with higher morbidity and mortality worldwide. Therefore, the current study was planned to assess the prevalence and risk factors of type-2 DM in Ethiopian population. Six electronic databases such as: PubMed, Scopus, Hinari, Web of science, Google Scholar, and African Journals Online were searched for studies published in English up December 30, 2020. Newcastle–Ottawa Scale was used for quality assessment of the included studies. The data was extracted by Microsoft excel and analyzed through Stata version 16 software. The random effect meta-regression analysis was computed at 95% CI to assess the pooled prevalence and risk factors of type-2 DM. Forty observational studies were included in this systematic review and meta-analysis. The pooled prevalence of DM in Ethiopia was 6.5% (95% CI (5.8, 7.3)). The sub-group analysis revealed that the highest prevalence of DM was found in Dire Dawa city administration (14%), and the lowest prevalence was observed in Tigray region (2%). The pooled prevalence of DM was higher (8%) in studies conducted in health facility. Factors like: Age ≥ 40 years ((Adjusted Odds Ratio (AOR): 1.91 (95% CI: 1.05, 3.49)), Illiterate (AOR: 2.74 (95% CI: 1.18, 6.34)), Cigarette smoking (AOR: 1.97 (95% CI: 1.17, 3.32)), Body mass index (BMI) ≥ 25 kg/m^2^ (AOR: 2.01 (95 CI: 1.46, 2.27)), family history of DM (AOR: 6.14 (95% CI: 2.80, 13.46)), history of hypertension (AOR: 3.00 (95% CI: 1.13, 7.95)) and physical inactivity (AOR: 5.79 (95% CI: 2.12, 15.77)) were significantly associated with type-2 DM in Ethiopian population. In this review, the prevalence of type-2 DM was high. Factors like: Older age, illiteracy, cigarette smoking, MBI ≥ 25, family history of DM, history of hypertension and physical inactivity were an identified risk factors of type-2 DM. Therefore, health education and promotion will be warranted. Further, large scale prospective studies will be recommended to address possible risk factors of type-2 DM in Ethiopian population.

## Introduction

Diabetes mellitus (DM) is a category of metabolic disorders characterized by hyperglycemia, which occurs when the pancreas stops producing enough insulin or when the body cannot use it. Diabetes causes a slew of complications that are linked to increased morbidity and mortality^1,2^. Diabetic Mellitus (DM) was prevalent in 9.3% of the world's population^3^. The mortality rate due to diabetes mellitus decreased from 2000 to 2010, then increased from 2010 to 2016 in the developed world, while the mortality rate due to DM increased in low-income countries during both periods^4^. The rise in the prevalence and incidence of diabetes has been attributed to changes in urban habits, which have been linked to sedentary behaviour^5^. In the next two decades, countries in Sub-Saharan Africa are predicted to see a significant rise in diabetes patients^6^. The prevalence of diabetes in Sub-Sahara Africa was reported to be lower (3%) than the global prevalence (8.5%) in 2016. However, undiagnosed diabetes mellitus in Africa (66.7%) is almost two times higher than that of developed countries 37%^7,8^, which contributes to the higher burden of morbidity and mortality in Africa.

Currently, Ethiopia has been challenged by the growing magnitude of non-communicable diseases (NCDs) such as diabetes. In Ethiopia, national data on the prevalence and incidence of diabetes are lacking. However, patient’s attendances and admission rates due to diabetes mellitus are rising in hospitals. In the previous 2–3 decades, there has been observable lifestyle changes with significant population growth and urbanization, which are the main risk factors repeatedly reported. In Ethiopia, according to the national WHO steps survey of 2015, the prevalence of diabetes mellitus was 3.2%^9^. A few other studies in Ethiopia report the prevalence of diabetes mellitus ranges from 0.5 to 6.5%^10,11^.

Even though in Ethiopia, one systematic review was published by Bishu.*et.al;* on the prevalence of diabetes mellitus and reported 2–6.5% prevalence of DM^12^. The previous review did not deal with socio-demographic risk factors that have a high potential effect on the development of diabetes. The review was done based on both type-1 and type-2 DM. Furthermore, the previous review did not provide evidence on the overall prevalence of DM nationwide and did not exhaustively include potential socio-demographic and personal characteristics in investigating their research. Therefore, this systematic review and meta-analysis study was aimed to assess predictors for the prevalence of type-2 diabetes mellitus in Ethiopia. The review study included previously conducted researches with different and few covariates considered as risk factors. The results obtained in the current investigation would significantly contribute to health professionals in delivering health-related education, care provision and further necessary for policy implication.

## Materials and methods

### Review strategies and inclusion criteria

A systematic review and meta-analysis were conducted to assess the pooled prevalence and the risk factors of type-2 DM in Ethiopia. An electronic search was conducted to retrieve studies. Published articles of cross-sectional, case–control and cohort studies were included. Databases PubMed, Hinari, Scopus, web of science, Google Scholar, and African journal online search engine were used to access the journals and articles published up to December 30, 2020. The search used the following medical subject heading terms; prevalence, risk factors, type-2 diabetes mellitus (DM) and Ethiopia separately or in combination. Preferred Reporting Items for Systematic Reviews and Meta-Analyses (PRISMA) guideline was utilized to conduct this review^13^.

#### Inclusion criteria

**Figure 1:**
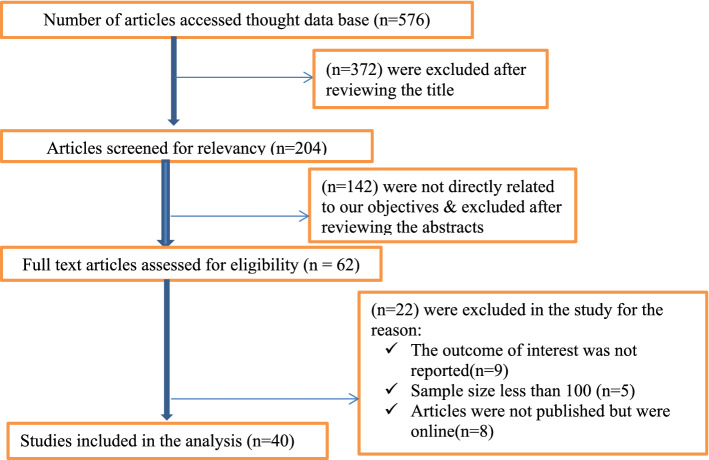
PRISMA flow diagram describing the selection of studies included in the systematic review and meta-analysis of prevalence and determinants of DM in Ethiopia.

Studies conducted in Ethiopia about prevalence and predictors of type-2 diabetes mellitus with a sample size of at least 100 observations, those had been reported in English language, and articles published, and available online were included for this review study. Among many of the previously published articles, only 40 satisfied the selection criteria (Fig. 1).

#### Study selection and screening

All citations identified by our search strategy were exported to EndNote-X9, and duplicate articles were removed. And then, the titles and abstracts of the identified articles were screened by two independent reviewers (MAZ and AAM) and eligible studies were included for further review. The full texts of selected articles were retrieved and read thoroughly to ascertain their suitability before data extraction. In case of disagreement between the two reviewers, the discussion has been held to reach a consensus and the third reviewer as consulted. The search process was presented in the PRISMA flow chart that clearly indicates the studies included and excluded with reasons of exclusion (Fig. 1)^13^.

#### Definition of outcome measures

The primary outcome of the study was the prevalence and risk factors of DM such as sex, age, family history of DM, body mass index (BMI), education status, cholesterol level, hypertension and we developed data collection tools to collect essential data from the studies included.

#### Risk factor

A condition, behavior or other factor that increases a person's chances of developing a disease.

#### Debates mellitus

The red blood cells are separated from the sample, and the amount of glucose is measured in the remaining plasma. A plasma level of 7.8 mmol/l (200 mg/l) or greater can indicate diabetes.

#### Type 2 diabetes mellitus

One of the two major types of diabetes mellitus, characterized by late age of onset (30 years or older), insulin resistance, high levels of blood sugar, and little or no need for supplemental insulin.

#### Quality assessment for studies

The quality of meta-analysis depends on the quality of the studies included^14^. Two authors (MAZ and ET) had assessed the risk of bias for the studies included using the modified version Newcastle–Ottawa Scale (NOS) for cross-sectional studies^15^. All items of the tool were filled in for each included study with the response of yes or no, and the quality of the study was determined by summing the score given for each item which rated as low bias ≥ 7 points, moderate bias three up to six points and high bias ≤ 3 points (Table 1). To increase the validity of this review, we included studies with low and moderate risk of bias^15^.

**Table 1:** Characteristics of the studies included and their prevalence of DM (N = 40).

Authors	Publication year	Study area (region)	Study design	Statistical model	Sample size	Response rate (%)	Prevalence of DM (%)	SE (%)	Quality assessment
Alemayehu et al.^16^	2018	Sidama	CS	OLR	2670	100	1.9	0.26	8 points
Endris et al.^17^	2020	SNNRP	CBCS	MVLR	634	98.89	5.7	0.92	8 points
Toyba et al.^18^	2019	Amhara	CBCS	MVL	587	100	6.8	1.03	6 points
Shiferaw et al.^19^	2018	Oromiya	CBCS	OLR	402	100	6.5	1.23	8 points
Gizaw et al.^20^	2015	AA	RS	Chi-square	8048	NS	6.5	0.32	6 points
Belete et al.^21^	2019	AA	CS	MVLR	392	100	2.6	0.8	8 points
Tilahun et al.^22^	2007	Oromiya	CS	OLR	576	91.32	5.3	0.93	7 points
Mahteme et al.^23^	2016	Amhara	CS	MVLR	1314	100	8.3	0.76	6 points
Seifu et al.^24^	2020	Sidama	CBCS	MVLR	519	99.4	12.4	0.48	8 points
Alemu et al.^25^	2020	Harari	IBCS	MVLR	415	98	8.8	1.39	6 points
Yoseph et al.^26^	2013	Oromiya	IBCS	ULR	422	100	5	1.06	8 points
Temesgen and Alemu^27^	2019	Amhara	IBCS	ULR	422	96.68	8.8	1.4	7 points
Duguma et al.^28^	2020	Oromiya	CS	MVLR	271	NS	11.4	1.93	6 points
Abdulahi^29^	2019	Somali	CBCS	MVLR	525	100	8.57	1.22	6 points
Solomon et al.^30^	2014	Amhara	CS	ULR	1100	97.3	5.1	0.66	8 points
Addisu and Getabalew^31^	2020	AA	IBCS	MVLR	758	100	14.8	1.29	6 points
Wondemagegn et al.^32^	2017	Amhara	CBCS	MVLR	757	95.4	11	1.13	8 points
Tesfa et al.^33^	2016	Amhara	CS	Chi-square	385	95.3	0.34	0.57	7 points
Ataro et al.^34^	2018	Harari	IBCS	MVLR	425	95.9	7.1	1.24	8 points
Chanyalew and Alemayehu^35^	2017	Oromiya	CBCS	ULR	605	98.2	7.3	1.06	8 points
Tariku et al.^36^	2016	AA	CS	MVLR	1003	93.32	5	0.69	7 points
Lemba et al.^37^	2012	Ethiopia	CS	Chi-square	2153	96.4	6.5	0.53	8 points
Muche et al.^38^	2019	Amhara	CS	ULR	1110	92.5	13	1.01	8 points
Esayas et al.^39^	2011	Oromiya	CS	Chi-square	329	100	10	1.65	8 points
Getachew et al.^40^	2020	Tigray	IBRS	Chi-square	299,806	100	1.02	0.02	7 points
Abebe et al.^41^	2013	Amhara	IBCS	Chi-square	354,524	NS	4.4	0.03	6 points
Getasew et al.^9^	2019	Amhara	CBCS	MVLR	607	100	10.2	1.22	7 points
Tesfaye et al.^42^	2020	Oromiya	CBCS	ULR	915	95.8	3.1	0.57	7 points
Worku and Yeshaneh^43^	2017	Ethiopia	CBCS	MVLR	1472	95.5	3.3	0.46	8 points
Assefa et al.^30^	2014	Amhara	IBCS	MVLR	225	88.4	8.5	1.86	8 points
Yeromnesh et al.^44^	2015	Tigray	IBRS	NS	20,939	100	1.3	0.09	6 points
Gezahegn et al.^45^	2020	Oromiya	IBCS	MVLR	321	98.4	5.1	1.22	7 points
Desalegn et al.^46^	2015	Harari	CS	ULR	787	91	7	0.91	8 points
Fisseha and Senthil^47^	2018	Oromiya	IBRS	NS	22,277	100	1.43	0.08	6 points
Endashaw et al.^48^	2014	AA	IBCS	MVLR	120	100	15.8	3.33	8 points
Gebreegziabiher et al.^10^	2020	Tigray	CBCS	MVLR	321	98.8	9.3	1.62	7 points
Bantie et al.^49^	2019	Amhara	CBCS	MVLR	607	100	10.2	1.22	6 points
Worede et al.^9^	2017	Amhara	CBCS	MVLR	392	NS	12	1.64	6 points
Seyoum et al.^50^	1999	Tigray	CBCS	NS	890	97.6	3.7	0.87	8 points
Tenaye et al.^51^	2019	DD	CS	ULR	463	90.92	13.5	1.58	7 points

Acronyms: *AA* Addis Ababa, *CBCS* community-based cross sectional study, *CS* cross-sectional study, *DD* Dire Dawa, *DM* diabetes mellitus, *FBCS* facility based cross sectional study, *IBCS* institution based cross sectional study, *HBRS* hospital-based retrospective study, *RS* retrospective study, *NS* not specified, *OLR* ordinal logistic regression, *MVLR* multivariate logistic regression, *ULR* uni-variate logistic regression.

#### Data extraction

Relevant data from each article was extracted by two independent reviewers (MAZ and ET). The data extraction includes; author name, year of publication, design of the study, sample size, study population, area of the study (region), response rate, the prevalence of DM, sex, age, family history of DM, BMI, alcohol consumption, cigarette smoking, hypertension, physical activity, educational status, cholesterol level, wasting circumference.

#### Description of studies

The literature review with the chosen search terms firstly identified 576 articles. We selected 204 articles after reviewing the proximate of the title to the predefined objectives. After further reading the full-text of each article, only 62 articles were subjected to document review. Lastly, 40 articles met the inclusion criteria for this systematic review and the meta-analysis (Fig. 1).

#### Data processing and analysis

After extracting the data from all eligible studies, the data were entered, and data analysis was done using (STATA version 16). The random-effects model was used for estimating the overall pooled prevalence of diabetes mellitus and its main components. In a meta-analysis, one of the relevant issues is the problem of heterogeneity among studies. To assess the consistency of studies, we use I^2^ test statistics^52^. This test examines the hypothesis of all the included studies are evaluated the same effect. Consequently, since there was heterogeneity between the original studies (I^2^ = 97.1%, *p* < 0.001), a random effect model was needed. To account for between-study variance, a random effect meta-analysis with an estimation of DerSimonian and Laird method was used^53,54^. The presence of publication bias is also accessed; funnel plots and tests of Begg’s were used as suggested by different scholars^55^.

### Ethics approval and consent to participate

This research is a systematic review and a meta-analysis in which data were gathered from published articles from websites, and hence ethical approval has not been obtained from the Institutional Review Board.

## Results and discussion

### Review results

In this study, we have searched about 576 studies initially from September 2020 up to December 2020. The flow chart diagram represents the number of studies searched, study selection, number of studies included in the study that meet the inclusion criteria. The process of systematic literature retrieval and screening of the studies are presented in (Fig. 1). Finally, 40 studies were included in this meta-analysis and systematic review.

### Description of included studies

Among those all studies included in this systematic review and meta-analysis, 14 (35%) of the studies were community-based cross-sectional, 13 (32.5%) studies were cross-sectional, 9 (22.5%) were institution based cross-sectional, and the remaining 4 (10%) studies were institution based retrospective. According to the regional locations of included studies, 22 (30%) were conducted in Amhara region, 9 (22.5%) were conducted in Oromia region, 5 (12.5%) in Addis Ababa, 4 (710%) in Tigray region, 3 (7.5%) in Harari region, 2 (5%) were conducted at the national level, 2 (5%) in Dire Dawa city administration and 1 (2.5%) studies were included in each region of SNNRP, Sidama region and Somali. But we could not find any study from Benishangul Gumuz, Afar and Gambella. From the studies which were included in this meta-analysis were used different types of statistical approach or methods to address their objectives. Among those studies about 19 (47.5%) were used multivariate logistic regression, 9 (22.5%) were used uni-variate logistic regression, 6 (15%) were used chi-square test, 3 (7.5%) were used ordinal logistic regression while the remaining 3 (7.5%) were not apply any statistical model rather they only study based on descriptive statistics, which mean that they are not study about risk factors though statistical models about the effect of them onT2DM (Table 1).

Results from Table 2, show that all studies did not used similar risk factors to study about the prevalence of T2DM. According to the associated factor sex, the highest prevalence of T2DM among the studies included were 18.4% for male and 16.2% for female which was conducted by Addisu & Getabalew while the lowest prevalence was 1.2% for both male and female which were obtained from two different study results Gezahegn et al.and Alemayehu *et.al* respectively.

**Table 2:** Summary statistics for the prevalence of T2DM by risk factors for each study.

Author	Associated risk factors of T2DM															
	Sex (%)		Age in year (%)		BMI (%)		WC (%)		Educational status (%)		Blood pressure (%)		Smoking (%)		Alcohol use (%)	
	Male	Female	< 40	≥ 40	< 25	≥ 25	Low	High	Illiterate	Literate	< 140	≥ 140	Yes	No	Yes	No
Alemayehu et al.	2.4	1.2	1.1	3.1	1.6	5.8	–	–		–	1.2	2.3	–	–	–	–
Endris et al.	6.5	4.6	2.1	3.6	8.3	10.5	–	–	5.2	0.5	–	–	–	–	–	–
Shiferaw et al.	–	–	–	–	3.7	6.5	1.9	11.2	–	–	10.5	17.8	–	–	–	–
Gizaw et al.	2.3	1.4	–	–	–	–	–	–	–	–	1.7	2.2	–	–	–	–
Belete et al.	2.5	2.6	3	7.7	0.4	6.3	0.3	9.3	9.1	7.1	2.7	5.6	2.9	2.5	2.8	1.8
Tilahun et al.	–	–	–	–	–	–	–	–	–	–	–	–	–	–	–	–
Mahteme et al.	2.1	1.7	2.4	3.4	–	–	–	–	–	–	–	–	–	–	–	–
Seifu et al.	–	–	–	–	2.6	5.5	–	–	–	–	4.6	7.7	6.9	5.4	–	–
Alemu et al.	–	–	–	–					1.2	7.5	–	–	–	–	–	–
Yoseph et al.	3.5	1.3	1.4	3.6	1.44	3.5	0.9	4	–	–	–	–	4.3	0.5	4	0.9
Duguma et al.	4.4	7	5.2	6.3	7.1	2.9	5.2	6.3	8.6	2.2	2.2	9.2	10.3	1.1		
Abdulahi	3.8	4.8	3	5.5					10	22.6	3.1	5.5	–	–	–	–
Solomon et al.	1.7	2.6			0.6	1.2	1.1	1.5	–	–	–	–	–	–	1.6	1.4
Addisu and Getabalew	18.4	16.2	3.4	10.5	5.5	9.2	–	–	–	–	6.7	8	1.6	13.2	6.5	8.3
Tariku et al.	–	–	4.9	7.5	4.9	7.9	–	–	–	–	4.7	8.1	2.1	10.7	–	–
Muche et al.	–	–	8.6	3.2	1.7	10.1			2.9	8.9	–	–	–	–	–	–
Getasew et al.	2.9	1.6	0.7	3.9	1.9	2.6	2.1	2.5	–	–	–	–	–	–	3.5	1.2
Worku and Yeshaneh	3.4	3.2	9.7	6.3	2.4	19	–	–	3.2	13.3	5	10	–	–	2.6	6
Assefa et al.	–	–	3.1	9.1	–	–	5.7	6.5	–	–	3.1	9.1	1.3	10.9	–	–
Gezahegn et al.	1.2	3.7	1.3	3.8	–	–			2.2	2.8	–	–	–	–	–	–
Desalegn et al.	7.8	5.4	4.9	9.9	6.5	8.02	4.2	8.7			–	–	–	–	8.8	6.6
Endashaw et al.	1.9	4.2	3.6	2.6	2.9	3.3			1.6	4.6	–	–	–	–	–	–
Bantie et al.	–	–	–	–	–	–	2.1	3.3	2.5	3.8	–	–	–	–	–	–
Ataro et al.	10.2	5.7	3.5	11.1	6.1	9.3	5.4	8.3	–	–	5.1	20.4	13.3	6.6	–	–

The prevalence of T2DM was higher in older age (age ≥ 40) years as compared to the elder age (age < 40 years) in almost all studies included except the study finding of Worku & Yeshaneh which was contradict4 of the other finding. The overall prevalence of T2DM was about 5.95% for age of ≥ 40 years and 3.6% for the patient whose age was less than 40 years. Similarly BMI was positively associated with TDM with the average overall prevalence of T2DM was about 3.6% for person whose BMI < 25 kg/m^2^ while the prevalence of T2DM was about 6.98% for persons whose BMI ≥ 25 kg/m^2^.The mean prevalence of T2DM was higher in illiterate case which accounts about 8.19%. The association of T2DM and alcohol consumption was, an individual who uses alcohol was more exposed to T2DM as compared to the none-user one. It is also similar in smokers. The overall mean prevalence of T2DM was 7.1% among the smoker individuals and 4.26% among alcohol users. Furthermore, blood pleasure and wasting circumference were also associated with T2DM. Among the studies included, there was the prevalence of about 9.89% T2DM among person with blood pressure of ≥ 140 mmHg while 5.46% person with blood pressure of < 140 mmHg (Table 2).

### Prevalence of type-2 diabetes mellitus in the study area

**Figure 2:**
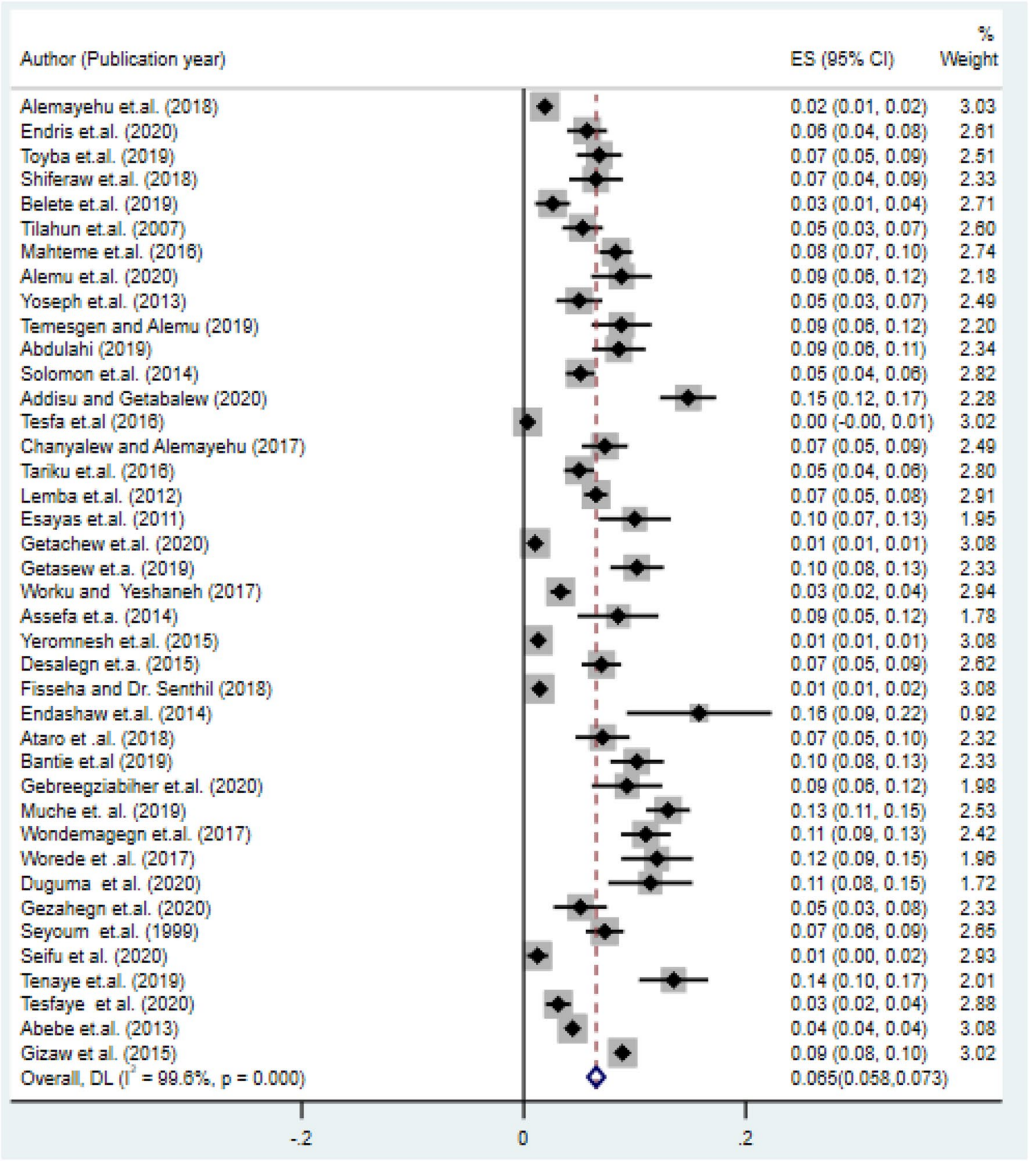
Forest plot of the pooled prevalence of diabetes mellitus in Ethiopia.

In this systematic review and meta-analysis, we observed a broader difference in the prevalence of type-2 DM among the included studies. The highest prevalence of type-2 DM was found in Addis Ababa (15.8%)^16^, while the lowest prevalence was confirmed in Amhara (0.34%)^33^. The overall pooled prevalence of type-2 diabetes in Ethiopia was 6.5% (95% CI :(5.8%, 7.3%)) (Fig. 2).

### Sub-group analysis of type-2 diabetes in Ethiopia

We had also conducted sub-group analyses to investigate how the prevalence of type-2 DM varies across different studies by region, study design, statistical method and data collection period. The sub-group analysis by region, the highest pooled prevalence of type-2 DM 14% (95% CI: 10%-17%) was in Dire Dawa city administration followed by 9% (95% CI: 6–11%) Addis Ababa, and the smallest were in Sidama 2% (1.1–2.4%). The sub-group analysis of type-2 DM by study design had shown the highest pooled prevalence, 8% (6–11%) for IBCS. Similarly, the highest magnitude of pooled prevalence of type-2 DM for sub-group analysis by study year was 7% (6–9%) in the studies conducted after 2016. This figure reflects that the current prevalence trend of type-2 DM has been increased as compared to the studies conducted before 2016. In the same explanation the highest prevalence of T2DM was observed from the study analyzed thought uni-variate logistic regression which was 7.4% (5.2–9.5%) followed by the chi-square test, 7% (3.1–10.8%).

Finally, since the value of I^2^ is large, implies that the heterogeneity on prevalence of T2DM was due to study area, study period, study design and also due to difference in statistical methodology (Table 3).

**Table 3:** Sub-group analysis of studies included in the meta-analysis on the prevalence and associated factors of DM in Ethiopia.

Sub-group	Random effects [95% CI]	Test of heterogeneity (I^2^) (%)
**By region**		
Amhara	8% [6–10%]	97.3
Oromiya	6% [4–8%]	94.4
Tigray	2% [2–3%]	96.7
Addis Ababa	9% [5–12%]	96.2
Sidama	2% [1.1–2.4%]	–
Harari	7% [6–9%]	–
Somali	9% [6–11%]	–
SNNRP	6% [4–8%]	–
National level	5% [2–8%]	95.1
Dire Dawa	14% [10–17%]	–
**By study design**		
CS	7% [5–9%]	97.1
CBCS	7% [5–9%]	93.4
IBCS	8% [6–11%]	92.5
IBRS	3% [2–4%]	99.5
**By study period**		
Before 2016	6% [4–7%]	99.2
After 2016	7% [6–9%]	97.4
**By statistical model**		
Chis-square	7% [3.1–10.8%]	96.6
MVLR	6.8% [4.9–8.8]	98.6
OLR	5.5% [1.6–9.4%]	95.9
ULR	7.4% [5.2–9.5% ]	97.4
NS	6.8% [2.7–10.8%]	94.5

### Risk factors of type-2 diabetes mellitus

In this systematic review and meta-analysis, we assessed the association of different associated risk factors like sex, age, body mass index, family history, hypertension, education status, use of alcohol, smoking habit, cholesterol level, and wasting circumference with type-2 DM in Ethiopia.

### Association of body mass index and hypertension with type-2 DM

**Figure 3:**
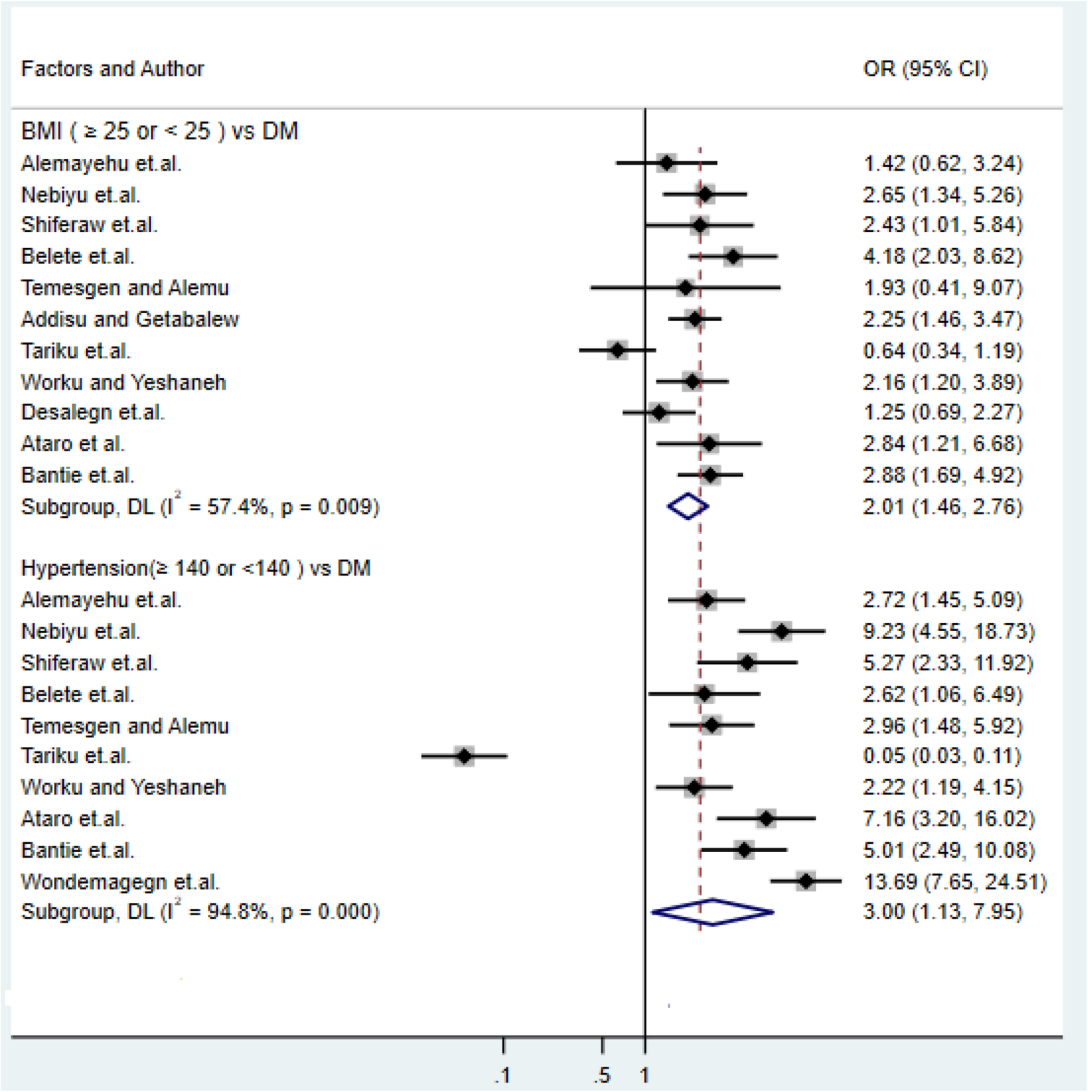
Forest plots of odds ratio for the association of body mass index and hypertension with type-2 DM.

In this sub-categorical meta-analysis, eleven studies were included to examine the effect of body mass index on type-2 DM^16,17,19,21,27,31,34,36,40,44,48,49^. Of these, seven studies indicated a statistically significant association between body mass index and type-2^17,19,21,31,34,43,49^ that shows a high risk of DM as body mass increased. In comparison, four studies showed there was no statistical difference in type-2DM among the normal weight and overweighed^17,27,36,46^. The pooled meta-regression analysis revealed a statistically significant difference in the exposure of type-2 DM among normal body mass index and obese with the odds ratio of 1.54 (95%: CI: 1.3, 1.83). Among the studies included in this meta-analysis, older age individuals were more exposed to type-2 diabetes than the younger age group, which is supported by the study^1,2,5,9,17,18,20,22–24,26,28,32,33,38,51,56^. In contrast, our study^11,23,35,45^ had got the result that contradict the other studies and concluded age had no any effect over the development of DM. Ten studies were examined the association between hypertension and DM^32,34,36,43,49^, and the result shows that a person with blood pressure ≥ 140 mm Hg was 2.48 times more likely to develop type-2 diabetes (OR: 2.48, 95% CI: 1.47, 4.18) (Fig. 3). Among those studies^16–20,27,32^, confirmed a significant statistical association between hypertension and type-2 DM.

### Association of age and cholesterol with type-2 DM

**Figure 4:**
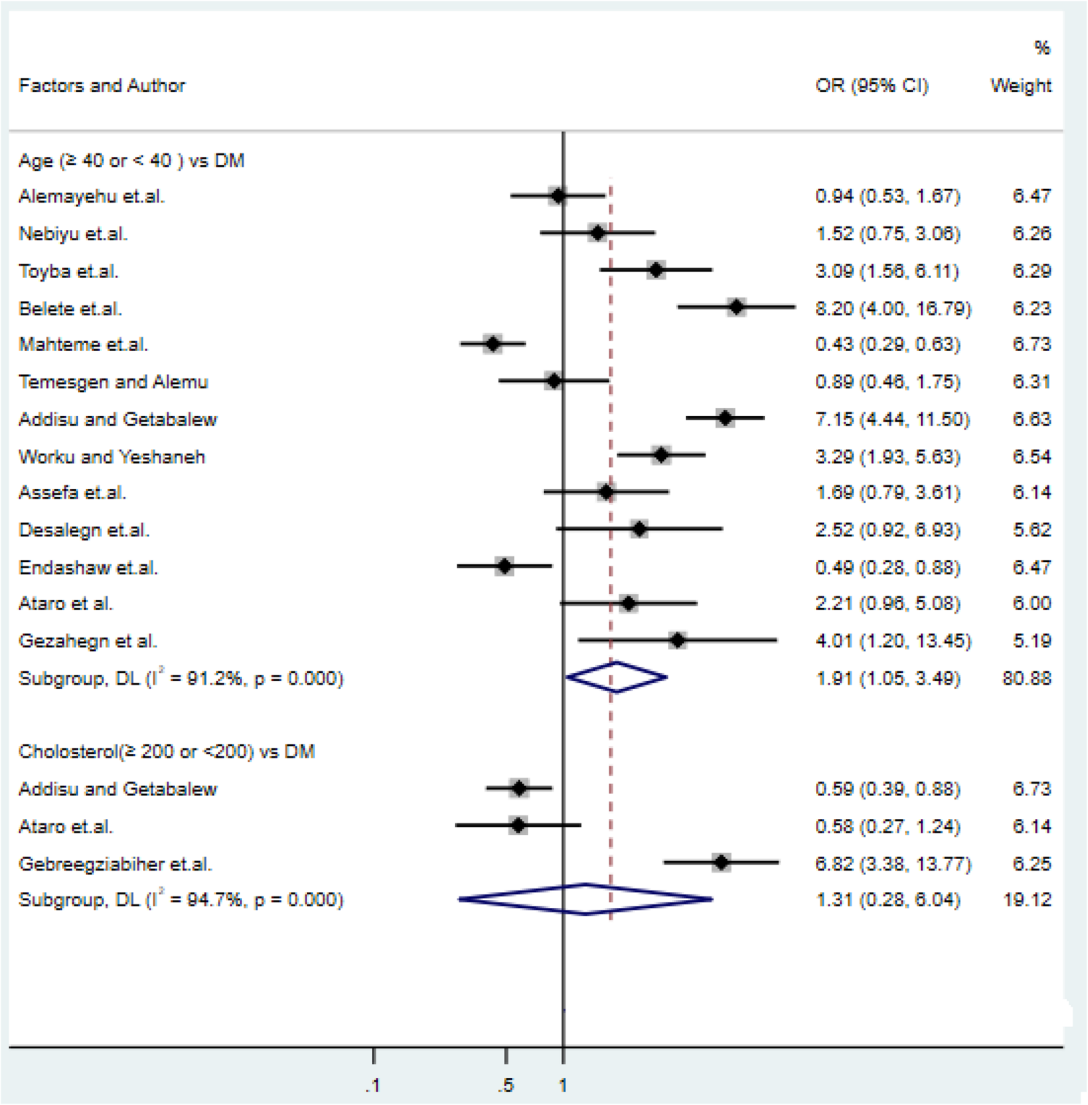
Forest plots of odds ratio for the association age and cholesterol with type-2 diabetes.

Thirteen studies had examined the association of age with type-2 DM^21,23,27,31,34,41,42,45,46,48^. Five of the studies had shown a statistically significant association of age with type-2 DM^18,21,31,43,45,48^ whereas the remaining seven studies have shown that age had no significant effect on type-2 DM. Consequently, the pooled meta-regression analysis showed that older age ≥ 40 years were statistical associated with type-2 DM (OR: 1.91, 95% CI: 1.05, 3.49), and cholesterol level ≥ 200 mg/dl was 1.31 times more likely to be exposed for diabetes (OR: 1.31, 95% CI: 0.28, 6.04) as compared cholesterol level < 200 mg/dl (Fig. 4) which is supported by the study of^10,31^.

### Association of education and physical exercise with type-2 DM

**Figure 5:**
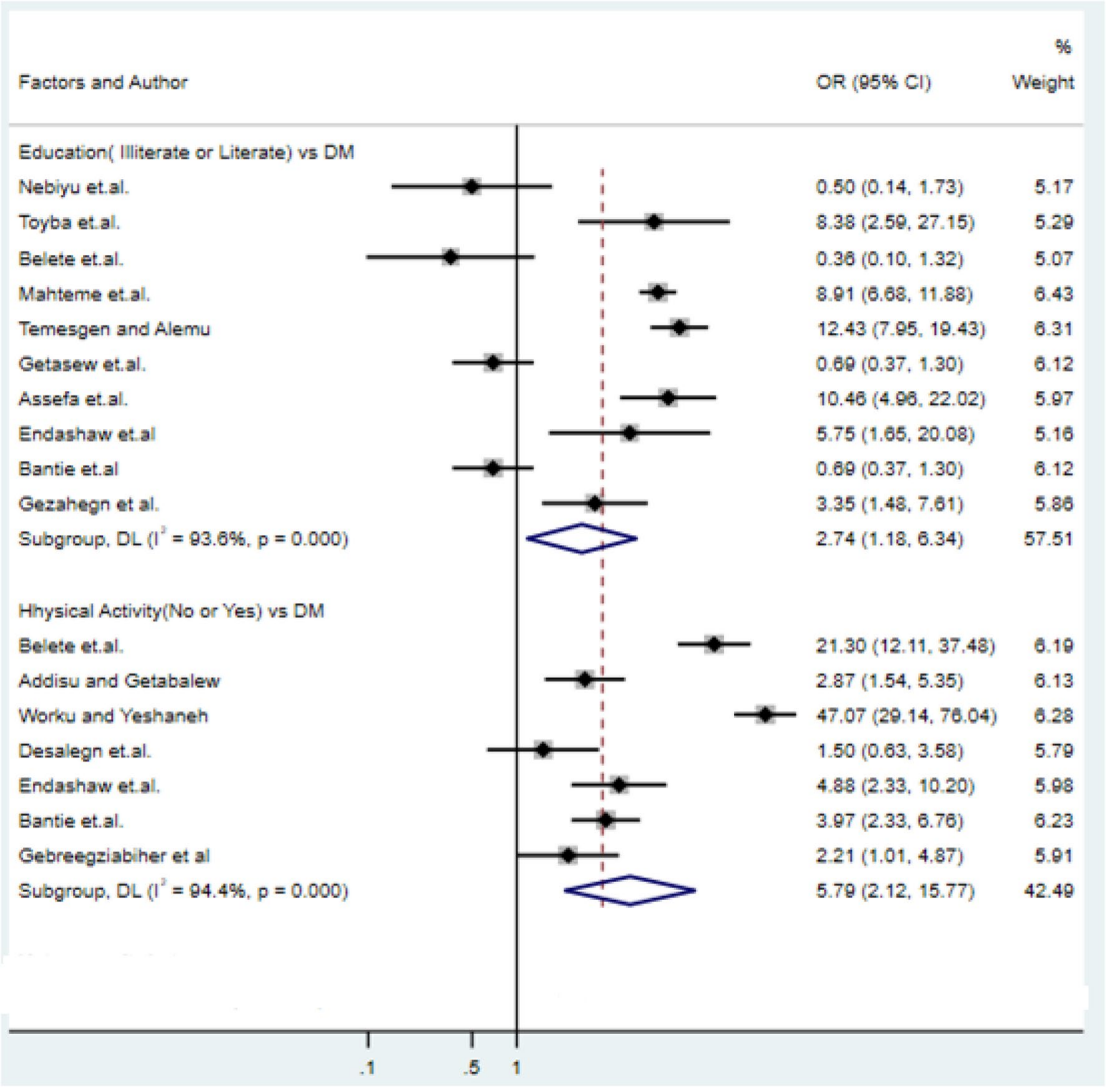
Forest plots of odds ratio for the association of education and physical activity with type-2 diabetes.

We use twelve studies to examine the association of education with DM and seven studies to investigate the association of physical activity with DM. Of 12 studies that examined the association of education level with type-2 DM, five studies supported that education level was associated with type-2 DM^11,45,48^. Among seven studies included in the analysis, six studies^10,43,47–49^ showed that physical activity had a significant contribution to the prevalence of type-2 DM (Fig. 5).

### Association of alcohol consumption, cigarette smoking and wasting circumference with type-2 DM

**Figure 6:**
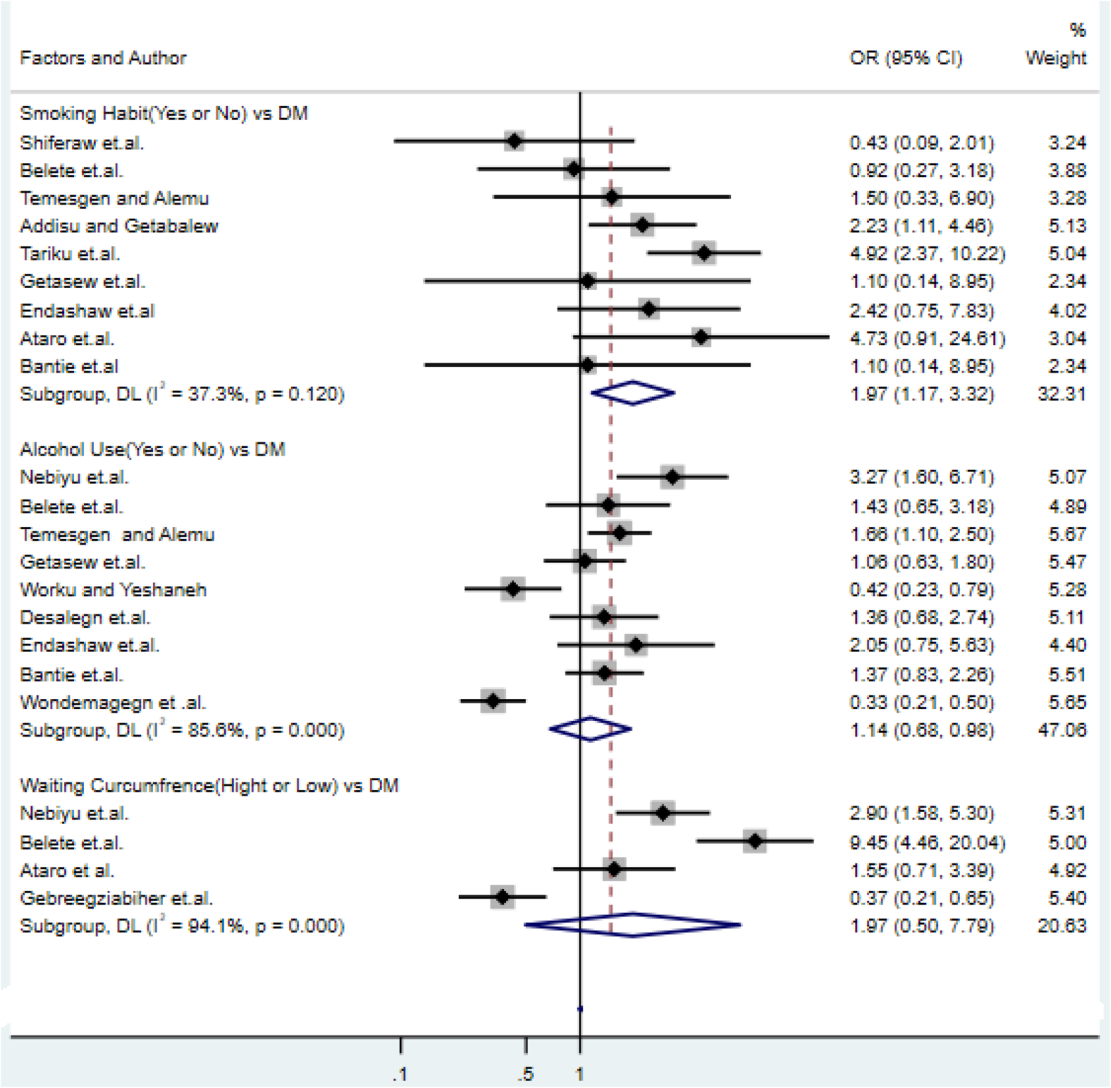
Forest plots of odds ratio for the association of smoking, alcohol consumption and wasting circumference with type-2 diabetes.

About the factor alcohol consumption studies^32,43^ with odds ratio of 1.14 (95% CI: 0.68, 0.98) and for factor cigarette smoking studies^31,36^ with an odds ratio of 1.97 (95% CI: 1.05, 7.79), showed alcohol consumption and cigarette smoking were significantly increase the risk of type-2 DM respectively. Moreover, another important vital factor this review that is a persistent association with type-2 DM, was wasting circumference. Wasting circumference (WC) is a diabetes risk factor that is strongly linked to other cardiovascular diseases. Study^10,49^ supports that an individual with high waist circumference was positively associated with DM and 1.97 times more likely to develop type-2 DM than the lower one(OR = 1.97, 95% CI: 1.05, 7.79) (Fig. 6).This result is also supported by the result of previous studies^26,57,58^.

### Association of family history of DM with type-2 DM

**Figure 7:**
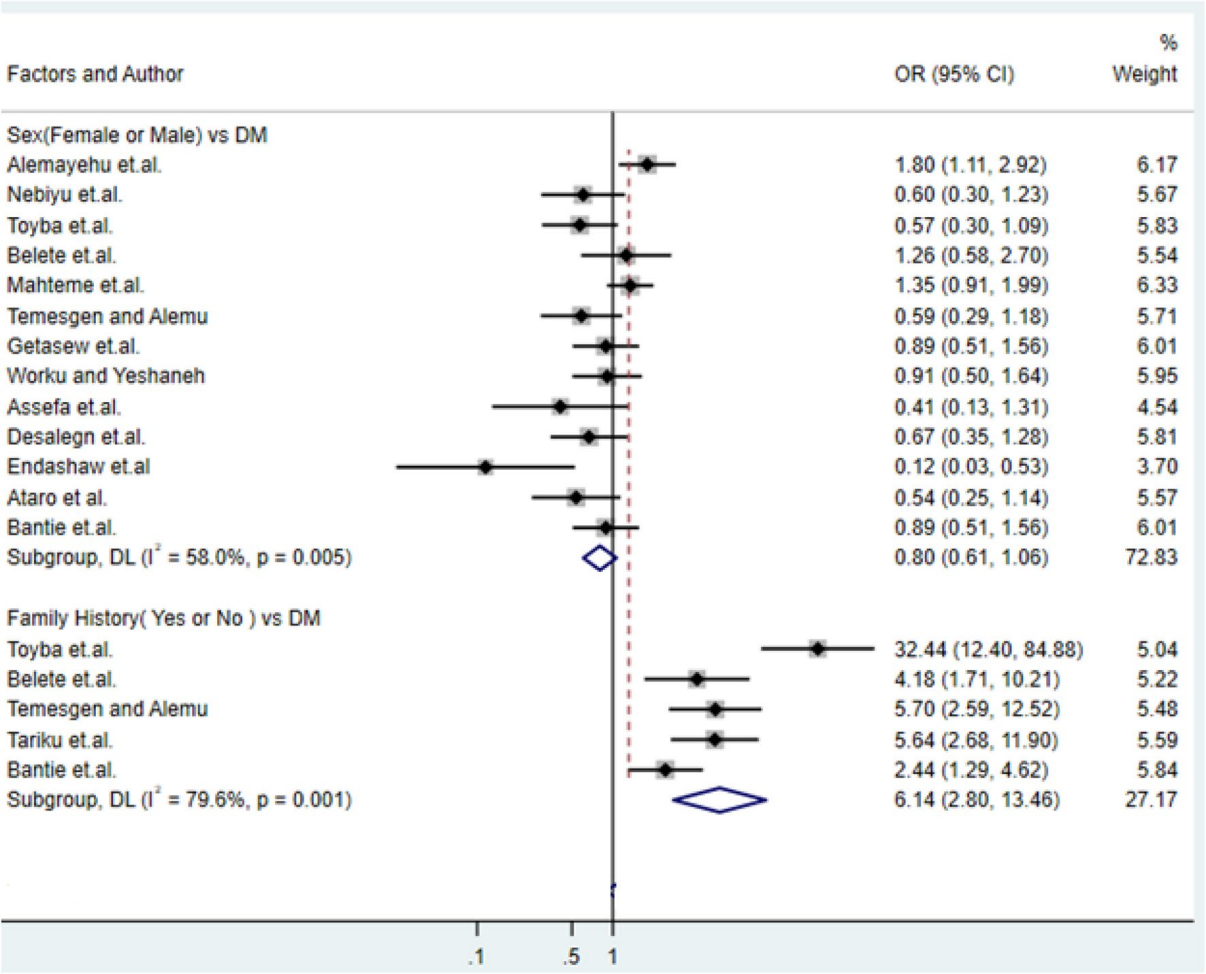
Forest plots of odds ratio for the association of sex and family history with type-2 diabetes.

Furthermore, among the studies included, about the variable family history study^18,21,27,36^ reflect having family history DM disease will increase the risk of type-2 DM with the odds ratio of 6.14 (95% CI: 2.8, 13.46) (Fig. 7).

### Risk of publication bias

**Figure 8:**
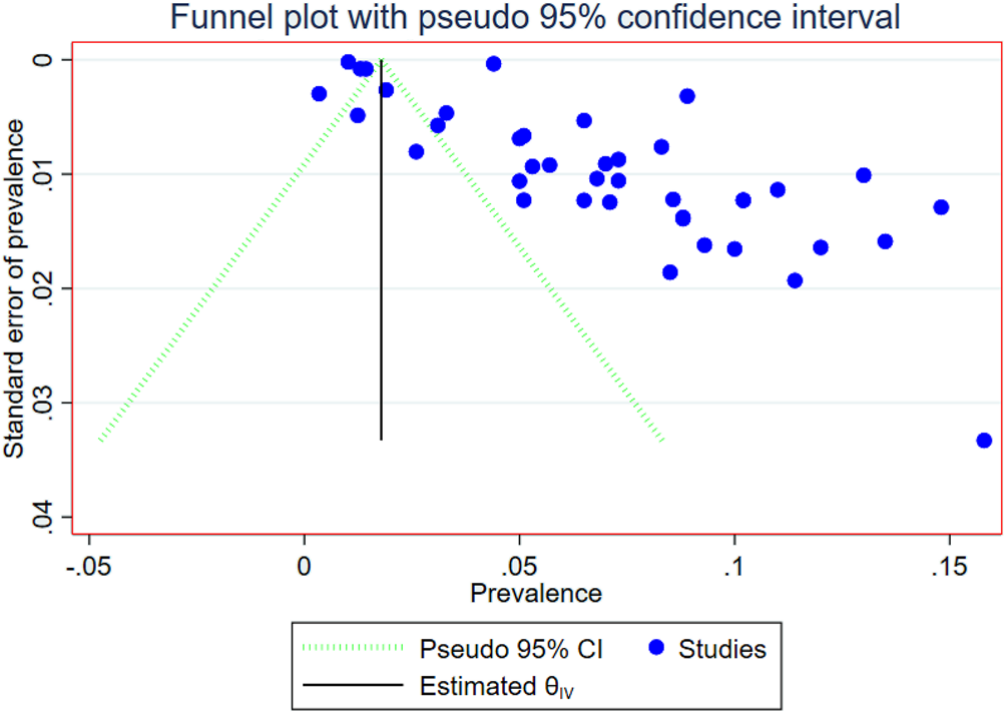
Funnel plot depicts publication bias of included studies on prevalence of DM in Ethiopia.

The issue of publication bias was assessed by visual inspection of the funnel plot and using Begg’s regression test. The funnel plot showed asymmetrical, and most studies are outside of the Pseudo 95% confidence interval with Begg’s test (*p* value = 0.176); both the plot and the p-value shows the existence of publication bias(Fig. 8). This bias may be the inclusion of different articles with different study designs, study population, study period and area for assessment of associated factors of DM. Sub-group analysis was done to further investigate the cause of heterogeneity, and the result was presented in (Fig. S1).

## Discussion

Non-communicable diseases are becoming a double burden of public health problems in developing countries, including Ethiopia. Besides, the prevalence of type-2 DM is rising in developing countries in contrast to developed nations. This systematic review and meta-analysis will, update pooled estimates of type-2 DM in Ethiopia, which gives valuable information for policymakers, health planners, and the community. The prevalence of type-2 DM was high with inadequate awareness, treatment and control of type-2 DM in Ethiopia. A healthy lifestyle should be advocated to reduce the prevalence and increase awareness of risk factors, treatment, and prevention of type-2 DM in Ethiopia. The magnitude of DM prevalence in this systematic review and meta-analysis is consistent with a systematic review and meta-analysis conducted in Ghana (6.2)^59^, Russian (6.4%)^60^, New Zealand (6%)^61^, and Taiwan (6.7)^62^. However, the magnitude of DM prevalence in this systematic review and meta-analysis is higher than the study result in Nigeria (3%)^56^. In comparison, the magnitude is lower than the study results in Beijing, China (10.75%)^63^, Africa (7.2%)^64^, Thailand (16.8%)^65^, Germany (14%)^66^, United States (22.1%)^67^ and Belgium (9.4%)^68^. The potential reason for this difference might be due to the different lifestyle of populations, a number of studies included life expectancy difference and the study settings.

In this study, there is a minimal difference in the pooled prevalence of type-2 DM among community-based cross-sectional and institution-based studies, which was 7.3% and 8.7%, respectively. This variation could be explained by institution-based studies that include DM patients who visit health facilities for medication.

A variety of variables were linked to type-2 diabetes in this systematic review and meta-analysis. As a result, in Ethiopia, age is a strong predictor of type 2 diabetes. Hence, people over the age of 40 are twice as likely as children to develop type-2 diabetes, and are more susceptible to infectious diseases, such as heart disease, lung disease, or kidney disease. This result is in line with the study conducted in Trinidad^69^.

Our review study results also found that physical activity related to daily living and commuting was as effective as leisure-time exercise in reducing the risk of type-2 DM. This result was agreed with the results obtained from the study conducted in China^70,71^. Alcohol consumption and the resulting health effect are more complex than the mere volume of consumption measured at one point in time. Additional alcohol measurements would add weight to the validity and relevance to the alcohol measure because it is long-term consumption that tends to be of medical and public health concern. Additionally, how alcohol is consumed (i.e. with meals or bingeing on weekends) affects various health outcomes. In this study, we found alcohol consumption is positively associated with type-2 DM. This result is supported by previously conducted^71,72^.

In the current study, type-2 diabetes was more common among diabetic patients who were illiterate. This difference is due to the fact that diabetes self-management education is critical for reducing weight, blood pressure, and alcohol intake, and literates should have a greater understanding and ability regarding feeding style in their dietary to avoid diabetes disease. This finding is consistent with the previous studies finding^46,47,49^.

We confirmed in this study that higher BMI is associated with increased insulin resistance and decreased insulin sensitivity in diagnosed type-2 DM. This study shows that higher BMI is the most important factor associated with type-2 DM in Ethiopian people. The potential reason might be that excess sugar can promote weight gain, thus type-2 DM through extra calories, but has no unique diabetogenic effect at physiological levels. This finding was consistent with conclusions from Italian and Japanese population studies^72,73^.

Furthermore, the current investigation also indicates that family history of DM, high waist circumference and hypertension (blood pressure) ≥ 140 mm Hg are more likely exposed to type-2 DM. This idea reflects that family history, waist circumference and hypertension (blood pressure) were significant risk factors of type-2 DM disease. This conclusion is in line with the conclusion of the study conducted in Trinidad population^70^.

Moreover, in this systematic review and meta-analysis, some studies found contradicted results about type-2 DM predictors. There were different possible reasons for the contradiction of results conducted by various researchers about a similar factor. The reasons for the contradicting results may be sample size difference, study design difference, the difference in the study population, study area and study time. Furthermore, studies have indicated that social support and self-efficacy are determinant factors of diabetes^59^.

### Strength and limitations of the study

#### Strength

This systematic examination and meta-analysis revealed the national pooled figure on DM risk factors in Ethiopia. Rising life expectancy, which is causing Ethiopia's population to age, is currently contributing to the rise in the risk of DM. As a result, the characteristics of type-2 DM prevalence as a function of population age and sex must be better understood. Owing to the unequal growth of regional economies in Ethiopia, type-2 diabetes is a modern disease that closely follows socio-economic development; it can be avoided and regulated by tailored interventions for different areas.

Finally, and perhaps most importantly, our research established several potentially useful prevention and control methods for type-2 diabetes. To minimize the prevalence of central obesity and associated type-2 diabetes, reasonable and safe diets, lifestyle improvements, cigarette smoking cessation, physical activity, and alcohol intake reduction are recommended.

#### Limitations

This review had not done without limitations. Firstly, the search strategy was limited to only published articles, but unpublished papers may be missed. Secondly, due to the inconsistent classification of factors for individual study, we cannot include all studies in pooled analysis quantitatively for the associated factors in this meta-analysis. This study was not without flaws. To begin with, the search strategy was limited to only the published articles; however, unpublished papers may have been overlooked. Second, we are unable to include all research in a pooled quantitative analysis for the related factors in this meta-analysis due to the inconsistent classification of factors for individual studies.

Finally, the clinical causes of type-2 diabetes mellitus were not included in this meta-analysis (HIV, TB, anemia, etc.).

## Conclusion

This systematic review and meta-analysis showed the national pooled figure on the risk factors of type-2 DM in Ethiopia. At present, increasing life expectancy, which leads to Ethiopia’s aging population, contributes to Ethiopia’s aging population and contributes to the increase in diabetes risk. Therefore, characteristics of type-2 DM prevalence with the changes in the population structure of age and sex need to be better understood. Type-2 DM is a modern disease that closely follows socio-economic development; it should be prevented and controlled by personalized measures for different areas due to the uneven development of regional economies in Ethiopia.

Finally and most importantly, our study identified some potentially effective prevention and control strategies to control the fast growth of type-2 DM. Reasonable and healthy diets use, lifestyle changes, stopping cigarette smoking, physical activity, and reducing alcohol consumption are recommended to reduce the occurrence of central obesity and subsequent type-2 DM.

To decrease the overall prevalence of type-2 DM in Ethiopia, we suggest that all physicians involved in diabetes mellitus diagnosis and treatment deliver tailored diabetes health education and strict counseling on self-management. We also recommend to the colleges, universities, ministry of science and higher education and ministry of health to train and deploy diabetes health educators in all relevant health facilities and promote physical exercises and enhancing social support will strongly help to overcome diabetes problem.

## Supplementary Information

Supplementary Figures.

Supplementary Information.

## Data Availability

Some of the data used for this study were attached with supporting material. All data were available from the corresponding author for reasonable requests.
