# Supplementary material for: Prevalence and risk factors of type-2 diabetes mellitus in Ethiopia: systematic review and meta-analysis
Source: Sci Rep. 2021 Nov 5;11:21733. doi: 10.1038/s41598-021-01256-9 (PMC8571297; doi:10.1038/s41598-021-01256-9)
Supplement: Supplementary file 1 — Supplementary Figures. [file 41598_2021_1256_MOESM1_ESM.docx]

**Supportive Graphs for Sub-group meta-analysis plot of the pooled prevalence of type-2 diabetes**


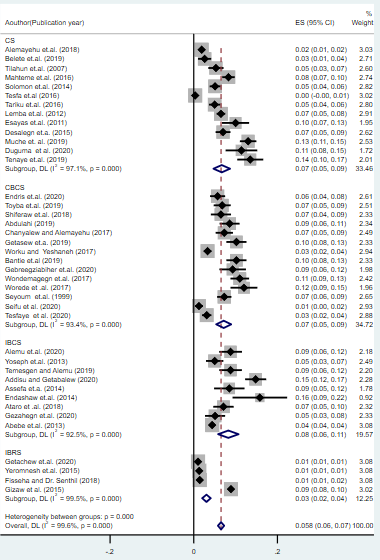


**Fig A. Forest plot of prevalence of type-2 diabetes by study design**


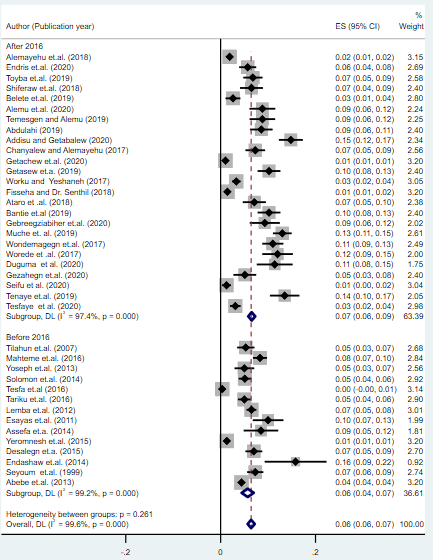


**Fig B. Forest plot of prevalence of type-2 diabetes by study period**


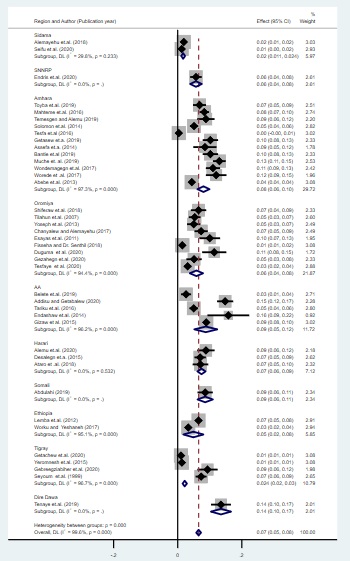


**Fig C. Forest plot of plod prevalence of T2DM by region**
