# Supplementary material for: Prevalence and risk factors of type-2 diabetes mellitus in Ethiopia: systematic review and meta-analysis
Source: Sci Rep. 2021 Nov 5;11:21733. doi: 10.1038/s41598-021-01256-9 (PMC8571297; doi:10.1038/s41598-021-01256-9)
Supplement: Supplementary file 2 — Supplementary Information. [file 41598_2021_1256_MOESM2_ESM.docx]

We used MESH key terms and a combination of these MeSH terms and free terms for the searching strategy to access studies included in this systematic review and meta-analysis.

**Some example of the searching strategy for the PubMed data base**

1. ((("Prevalence"[Mesh]) AND "Risk Factors"[Mesh]) AND "Diabetes Mellitus, Type- 2"[Mesh]) AND "Ethiopia"[Mesh]
2. ((("Prevalence"[Mesh]) OR "Risk Factors"[Mesh]) AND "Diabetes Mellitus, Type 2"[Mesh]) AND "Ethiopia"[Mesh]
3. ((((prevalence) AND (and)) AND (Associated Factors)) AND (Type-2 Diabetes)) AND (Ethiopia)
4. ((((prevalence) OR(or)) OR (Associated Factors)) AND (Type-2 diabetes)) AND (Ethiopia)
